# Hardware Design for Autonomous Bayesian Networks

**DOI:** 10.3389/fncom.2021.584797

**Published:** 2021-03-08

**Authors:** Rafatul Faria, Jan Kaiser, Kerem Y. Camsari, Supriyo Datta

**Affiliations:** ^1^Department of Electrical and Computer Engineering, Purdue University, West Lafayette, IN, United States; ^2^Department of Electrical and Computer Engineering, University of California, Santa Barbara, Santa Barbara, CA, United States

**Keywords:** Bayesian network, probabilistic spin logic, binary stochastic neuron, magnetic tunnel junction, inference

## Abstract

Directed acyclic graphs or Bayesian networks that are popular in many AI-related sectors for probabilistic inference and causal reasoning can be mapped to probabilistic circuits built out of probabilistic bits (p-bits), analogous to binary stochastic neurons of stochastic artificial neural networks. In order to satisfy standard statistical results, individual p-bits not only need to be updated sequentially but also in order from the parent to the child nodes, necessitating the use of sequencers in software implementations. In this article, we first use SPICE simulations to show that an autonomous hardware Bayesian network can operate correctly without any clocks or sequencers, but only if the individual p-bits are appropriately designed. We then present a simple behavioral model of the autonomous hardware illustrating the essential characteristics needed for correct sequencer-free operation. This model is also benchmarked against SPICE simulations and can be used to simulate large-scale networks. Our results could be useful in the design of hardware accelerators that use energy-efficient building blocks suited for low-level implementations of Bayesian networks. The autonomous massively parallel operation of our proposed stochastic hardware has biological relevance since neural dynamics in brain is also stochastic and autonomous by nature.

## 1. Introduction

Bayesian networks (BN) or belief nets are probabilistic directed acyclic graphs (DAG) popular for reasoning under uncertainty and probabilistic inference in real-world applications such as medical diagnosis (Nikovski, 2000), genomic data analysis (Friedman et al., 2000; Jansen et al., 2003; Zou and Conzen, 2004), forecasting (Sun et al., 2006; Ticknor, 2013), robotics (Premebida et al., 2017), image classification (Arias et al., 2016; Park, 2016), neuroscience (Bielza and Larrañaga, 2014), and so on. BNs are composed of probabilistic nodes and edges from *parent* to *child* nodes and are defined in terms of conditional probability tables (CPT) that describe how each *child node* is influenced by its *parent nodes* (Heckerman and Breese, 1996; Koller and Friedman, 2009; Pearl, 2014; Russell and Norvig, 2016). The CPTs can be obtained from expert knowledge and/or machine learned from data (Darwiche, 2009). Each node and edge in a BN have meaning representing specific probabilistic events and their conditional dependencies and they are easier to interpret (Correa et al., 2009) than neural networks where the hidden nodes do not necessarily have meaning. Unlike neural networks where useful information is extracted only at the output nodes for prediction purposes, BNs are useful for both prediction and inference by looking at not only the output nodes but also other nodes of interest. Computation of different probabilities from a BN becomes intractable when the network gets deeper and more complicated with child nodes having many parent nodes. This has inspired various hardware implementations of BNs for efficient inference (Rish et al., 2005; Chakrapani et al., 2007; Weijia et al., 2007; Jonas, 2014; Querlioz et al., 2015; Zermani et al., 2015; Behin-Aein et al., 2016; Friedman et al., 2016; Thakur et al., 2016; Tylman et al., 2016; Shim et al., 2017). In this article, we have elucidated the design criteria for an autonomous (clockless) hardware for BN unlike other implementations that typically use clocks.

Recently, a new type of hardware computing framework called probabilistic spin logic (PSL) is proposed (Camsari et al., 2017a) based on a building block called probabilistic bits (p-bits) that are analogous to binary stochastic neurons (BSN) (Ackley et al., 1985; Neal, 1992) of the artificial neural network (ANN) literature. p-bits can be interconnected to solve a wide variety of problems such as optimization (Sutton et al., 2017; Borders et al., 2019), inference (Faria et al., 2018), an enhanced type of Boolean logic that is invertible (Camsari et al., 2017a; Faria et al., 2017; Pervaiz et al., 2017, 2018), quantum emulation (Camsari et al., 2019), and *in situ* learning from probability distributions (Kaiser et al., 2020).

**Figure 1:**
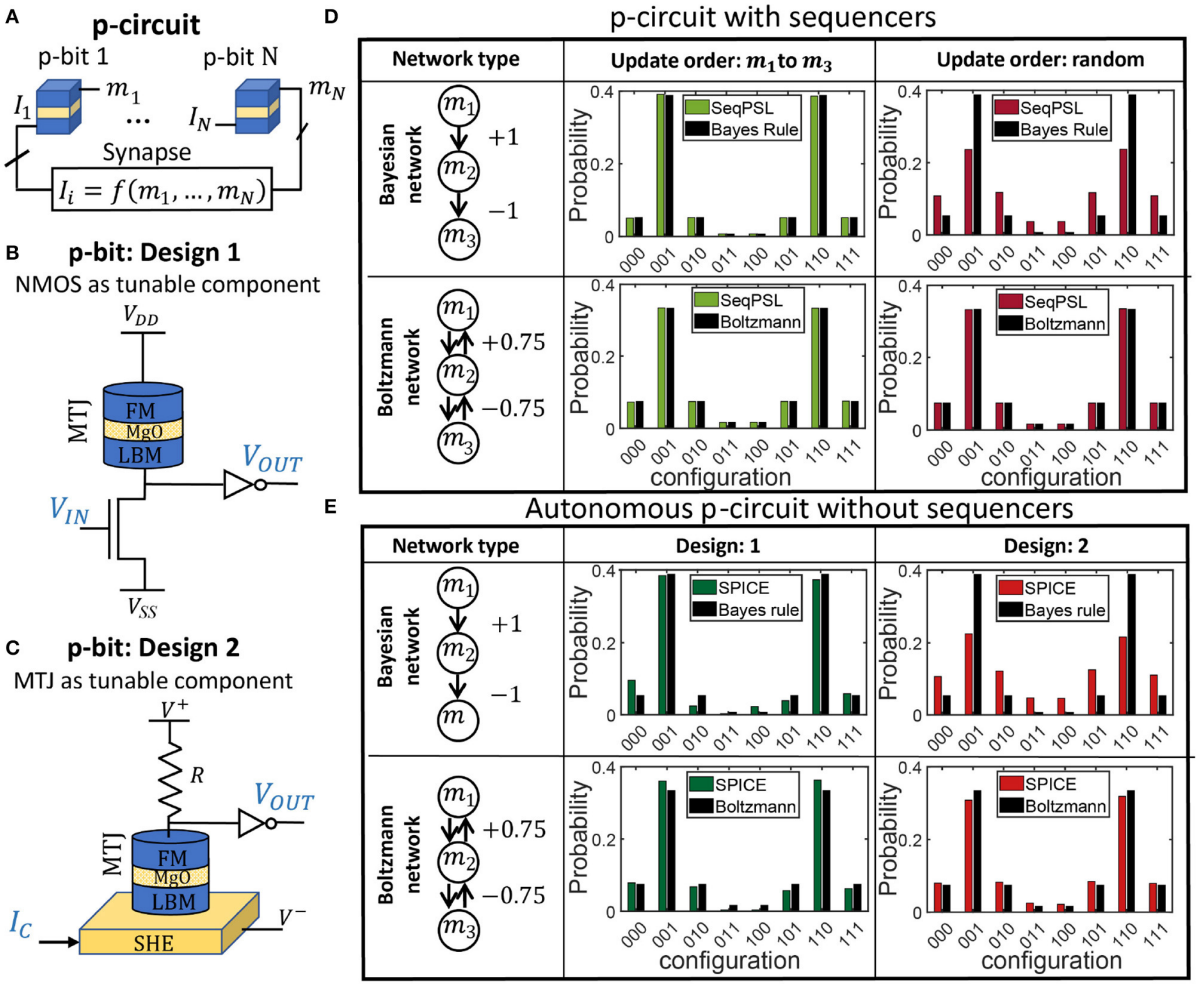
Clocked vs. autonomous p-circuit: **(A)** a probabilistic (p-)circuit is composed of p-bits interconnected by a weight logic (synapse) that computes the input *I*_*i*_ to the *i*^*th*^ p-bit as a function of the outputs from other p-bits. **(B)** p-bit design 1 based on stochastic Magnetic Tunnel Junction (s-MTJ) using low barrier nanomagnets (LBMs) and an NMOS transistor as tunable component. **(C)** p-bit design 2 based on s-MTJ as tunable component. Both designs have been used to build a p-circuit as shown in **(A)**. **(D)** Two types of p-circuits are built: a directed or Bayesian network and a symmetrically connected Boltzmann network. The p-circuits are sequential (labeled as SeqPSL) that means p-bits are updated sequentially, one at a time, using a clock circuitry with a sequencer. It is shown that for Boltzmann networks update order does not matter and any random update order would produce the correct probability distribution. But for Bayesian networks, a specific, parent-to-child update order is necessary to converge to the correct probability distribution dictated by the Bayes rule. **(E)** The same Bayesian and Boltzmann p-circuits are implemented on an autonomous hardware built with p-bit design 1 and 2 without any clocks or sequencers. It is interesting to note that for Bayesian networks, design 2 fails to match the probabilities from applying Bayes rule, whereas design 1 works quite well as an autonomous Bayesian network. For every histogram in this figure, 10^6^ samples have been collected.

Unlike conventional deterministic networks built out of deterministic, stable bits, stochastic or probabilistic networks composed of p-bits (Figure 1A), can be correlated by interconnecting them to construct p-circuits defined by two equations (Ackley et al., 1985; Neal, 1992; Camsari et al., 2017a): (1) a p-bit/BSN equation and (2) a weight logic/synapse equation. The output of a p-bit, *m*_*i*_, is related to its dimensionless input *I*_*i*_ by the equation:

(1a) $$\begin{array}{r} m_{i}\left(t+\tau_{N}\right)=\text{sgn}(\text{rand}\left(-1,1\right)+\text{tanh }I_{i}\left(t\right)) \end{array}$$

where rand(−1, +1) is a random number uniformly distributed between −1 and +1, and τ_*N*_ is the neuron evaluation time.

The synapse generates the input *I*_*i*_ from a weighted sum of the states of other p-bits. In general, the synapse can be a linear or non-linear function, although a common form is the linear synapse described according to the equation:

(1b) $$\begin{array}{r} I_{i}\left(t+\tau_{S}\right)=I_{0}(h_{i}+\underset{j}{\sum }J_{ij}m_{j}\left(t\right)) \end{array}$$

where *h*_*i*_ is the on-site bias and *J*_*ij*_ is the weight of the coupling from *j*^*th*^ p-bit to *i*^*th*^ p-bit, *I*_0_ parameterizes the coupling strength between p-bits, and τ_*S*_ is the synpase evaluation time. Several hardware designs of p-bits based on low barrier nanomagnet (LBM) physics have been proposed and also experimentally demonstrated (Ostwal et al., 2018; Borders et al., 2019; Ostwal and Appenzeller, 2019; Camsari et al., 2020; Debashis, 2020). The thermal energy barrier of the LBM is of the order of a few *k*_*B*_*T* instead of 40–60 *k*_*B*_*T* used in the memory technology to retain stability. Because of thermal noise the magnetization of the LBM keeps fluctuating as a function of time with an average retention time τ~τ_0_exp(*E*_*B*_/*k*_*B*_*T*) (Brown, 1979), where τ_0_ is a material-dependent parameter called attempt time that is experimentally found to be in the range of nanosecond or less and *E*_*B*_ is the thermal energy barrier (Lopez-Diaz et al., 2002; Pufall et al., 2004). The stochasticity of the LBMs makes them naturally suitable for p-bit implementation.

Figure 1 shows two p-bit designs: Design 1 (Figure 1B) (Camsari et al., 2017b; Borders et al., 2019) and Design 2 (Figure 1C) (Camsari et al., 2017a; Ostwal and Appenzeller, 2019). Designs 1 and 2 both are fundamental building blocks of spin transfer torque (STT) and spin orbit torque (SOT) magnetoresistive random access memory (MRAM) technologies, respectively (Bhatti et al., 2017). Their technological relevance motivates us to explore their implementations as p-bits. Design 1 is very similar to the commercially available 1T/1MTJ (T: Transistor, MTJ: Magnetic Tunnel Junction) embedded MRAM device where the free layer of the MTJ is replaced by an in-plane magnetic anisotropy (IMA) or perpendicular magnetic anisotropy (PMA) LBM. Design 2 is similar to the basic building block of SOT-MRAM device (Liu et al., 2012) where the thermal fluctuation of the free layer magnetization of the stochastic MTJ (s-MTJ) (Vodenicarevic et al., 2017, 2018; Mizrahi et al., 2018; Parks et al., 2018; Zink et al., 2018; Borders et al., 2019) is tuned by a spin current generated in a heavy metal layer underneath the LBM due to SOT effect. The in-plane polarized spin current from the SOT effect in the spin hall effect (SHE) material in design 2 requires an in-plane LBM to tune its magnetization, although a perpendicular LBM with a tilted anisotropy axis is also experimentally shown to work (Debashis et al., 2020). However, design 2 requires spin current manipulation, design 1 does not rely on that as long as circular in-plane LBMs with continuous valued magnetization states that are hard to pin are used. In-plane LBMs also provide faster fluctuation than perpendicular ones leading to faster sampling speed in the probabilistic hardware (Hassan et al., 2019; Kaiser et al., 2019).

**Figure 2:**
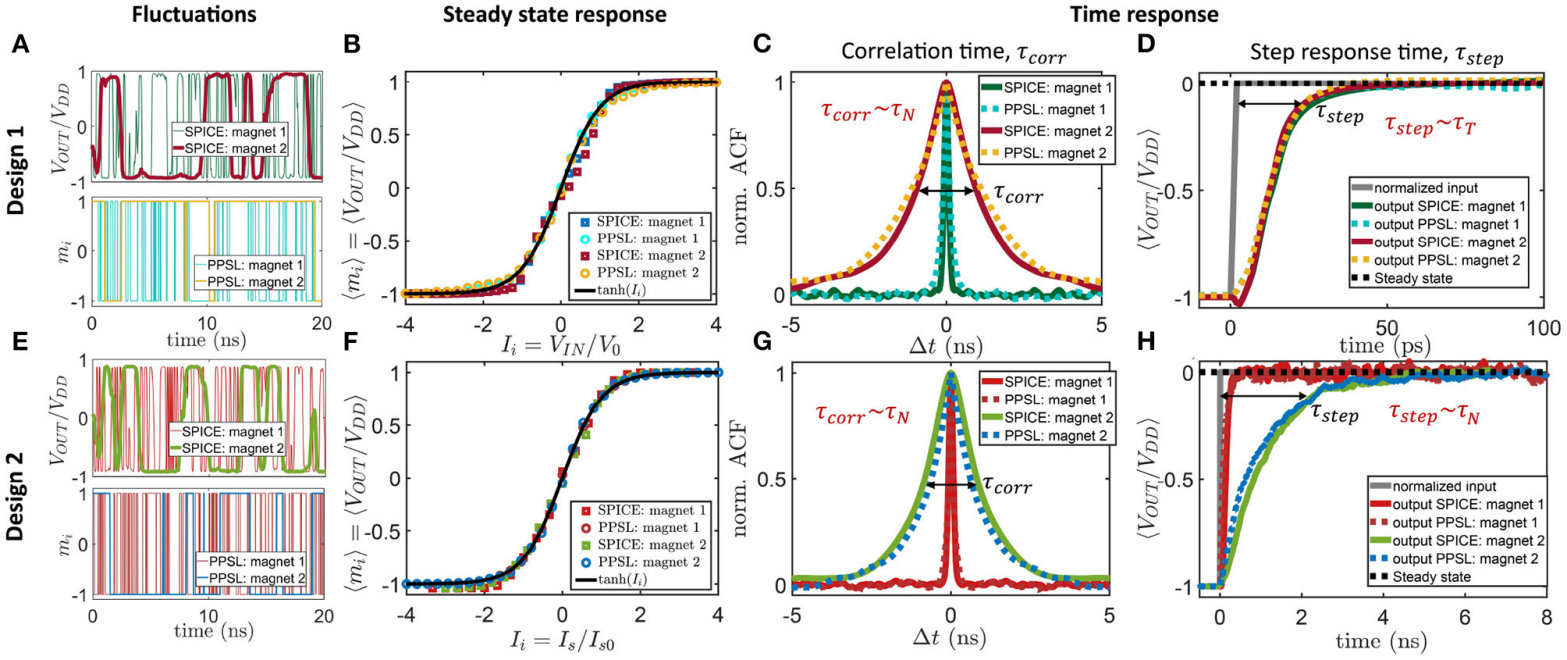
Autonomous behavioral model for p-bit: **(A–D)** Behavioral model for the autonomous hardware with design 1 (Figure 1B) is benchmarked with SPICE simulations of the actual device involving experimentally benchmarked modules. The behavioral model (labeled as “PPSL”) shows good agreement with SPICE in terms of capturing fluctuation dynamics **(A)**, steady-state sigmoidal response **(B)**, and two different time responses: autocorrelation time of the fluctuating output under zero input condition labeled as τ_*corr*_ **(C)**, which is proportional to the LBM retention time τ_*N*_ in the nanosecond range, and the step response time τ_*step*_ **(D)** that is proportional to transistor response time τ_*T*_, which is few picoseconds and much smaller than τ_*N*_. The magnet parameters used in the simulations are mentioned in section 2. **(E–H)** Similar benchmarking for p-bit design 2 (Figure 1C). In this case, τ_*step*_ is proportional to τ_*N*_. For **(B,F)**, each point for the SPICE simulation was obtained by averaging *m*_*i*_ over 1 μs. The step response time for **(D,H)** is obtained by averaging over 2,000 ensembles where *I*_*i*_ = −5 at *t* < 0 and *I*_*i*_ = 0 at *t* > 0.

The key distinguishing feature of the two p-bit designs (designs 1 and 2) is the time scales in implementing Equation (1a). From a hardware point of view, Equation (1a) has two components: a random number generator (RNG) (rand) and a tunable component (tanh). In design 1, the RNG is the s-MTJ utilizing an LBM and the tunable component is the NMOS transistor, thus having two different time scales in the equation. But in design 2, both the RNG and the tunable component are implemented by a single s-MTJ utilizing an LBM, thus having just one time scale in the equation. This difference in time scales in the two designs is shown in Figure 2. Note that although the two p-bit designs have the same RNG source, namely a fluctuating magnetization, it is the difference in their circuit configuration with or without the NMOS transistor in the MTJ branch that results in different time dynamics of the two designs.

In traditional software implementations, p-bits are updated sequentially for accurate operation such that after each τ_*S*_+τ_*N*_ time interval, only one p-bit is updated (Hinton, 2007). This naturally implies the use of sequencers to ensure the sequential update of p-bits. The sequencer generates an *Enable* signal for each p-bit in the network and ensures that no two p-bits update simultaneously. The sequencer also makes sure that every p-bit is updated at least once in a time step where each time step corresponds to *N*·(τ_*S*_+τ_*N*_), *N* being the number of p-bits in the network. (Roberts and Sahu, 1997; Pervaiz et al., 2018). For symmetrically connected networks (*J*_*ij*_ = *J*_*ji*_) such as Boltzmann machines, the update order of p-bits does not matter and any random update order produces the standard probability distribution described by equilibrium Boltzmann law as long as p-bits are updated sequentially. But for directed acyclic networks (*J*_*ij*_≠0, *J*_*ji*_ = 0) or BNs to be consistent with the expected conditional probability distribution, *p-bits need to be updated not only sequentially but also in a specific update order, which is from the parent to child nodes* (Neal, 1992) similar to the concept of forward sampling in belief networks (Henrion, 1988; Guo and Hsu, 2002; Koller and Friedman, 2009). As long as this parent to child update order is maintained, the network converges to the correct probability distribution described by probability chain rule or Bayes rule. This effect of update order in a sequential p-circuit is shown on a three p-bit network in Figure 1D. In the Supplementary Material, it is shown in an example how the CPT of the BN can be mapped to a p-circuit following Faria et al. (2018).

Unlike sequential p-circuits in ANN literature, the distinguishing feature of our probabilistic hardware is that it is *autonomous* where each p-bit runs in parallel without any clocks or sequencers. This autonomous p-circuit (ApC) allows massive parallelism potentially providing peta flips per second sampling speed (Sutton et al., 2020). The complete sequencer-free operation of our “autonomous” p-circuit is very different from the “asynchronous” operation of spiking neural networks (Merolla et al., 2014; Davies et al., 2018). Although p-bits are fluctuating in parallel in an ApC, it is very unlikely that two p-bits will update at the exact same time since random noise control their dynamics. Therefore, persistent parallel updates are extremely unlikely and are not a concern. Note that even if p-bits update sequentially, each update has to be *informed* such that when one p-bit updates it has received the up-to-date input *I*_*i*_ based on the latest states of other p-bits *m*_*j*_ that it is connected to. This informed update can be ensured as long as the synapse response time is much faster than the neuron time (τ_*S*_ ≪ τ_*N*_) and this is a key design rule for an ApC. If the input of the p-bit is based on old state of neighboring p-bits or on time-integrated synaptic inputs, the ApC operation declines in functionality or fails completely. However, for τ_*S*_ ≪ τ_*N*_, the ApC works properly for a Boltzmann network without any clock because no specific update order is required in this case. But, it is not intuitive at all if an ApC would work for a BN because a particular parent to child *informed* update order is required in this case, as shown in Figure 1D. As such, it is not straightforward that a clockless autonomous circuit can naturally ensure this specific informed update order. In Figure 1E, we have shown that it is possible to design hardware p-circuit that can naturally ensure a parent to child informed update order in a BN without any clocks. In Figure 1E, two p-bit designs are evaluated for implementing both Boltzmann network and BN. We have shown that design 1 is suitable for both Boltzmann network and BN. But design 2 is suitable for Boltzmann networks only and does not work for BNs in general. The synapse in both types of p-circuits is implemented using a resistive crossbar architecture (Alibart et al., 2013; Camsari et al., 2017b), although there are also other types of hardware synapse implementations based on memristors (Li et al., 2018; Mahmoodi et al., 2019; Mansueto et al., 2019), magnetic tunnel junctions (Ostwal et al., 2019), spin orbit torque driven domain wall motion devices (Zand et al., 2018), phase change memory devices (Ambrogio et al., 2018), and so on. In all the simulations, τ_*S*_ is assumed to be negligible compared to other time scales in the circuit dynamics.

Our proposed probabilistic hardware for BNs shows significant biological relevance because of the following reasons: (1) The brain consists of neurons and synapses. The basic building block called “p-bit” of our proposed hardware mimics the neuron and the interconnection among p-bits mimics the synapse function. (2) The components of brain are stochastic or noisy by nature. p-bits mimicking the neural dynamics in our proposed hardware are also stochastic. (3) Brain does not have a single clock for synchronous operation and can perform massively parallel processing (Strukov et al., 2019). Our autonomous hardware also does not have any global clock or sequencers and each p-bit fluctuates in parallel allowing massively parallel operation.

Further, we have provided a behavioral model in section 2 for both designs 1 and 2, illustrating the essential characteristics needed for correct sequencer-free operation of BNs. Both models are benchmarked against state-of-the-art device/circuit models (SPICE) of the actual devices and can be used for the efficient simulation of large-scale autonomous networks.

## 2. Behavioral Model for Autonomous Hardware

In this section, we will develop an autonomous behavioral model that we will call parallel probabilistic spin logic (PPSL) for design 1 (Figure 1B) and revisit the behavioral model for design 2, which was proposed by Sutton et al. (2020). The term “Parallel” refers to all the p-bits fluctuating in parallel without any clocks or sequencers. These behavioral models are high-level representations of the p-circuit and p-bit behavior and connect Equations (1a) and (1b) to the hardware p-bit designs. Please note the parameters introduced in these models will represent certain parts of the p-bit and synapse behavior like MTJ resistances (*r*_*MTJ*_) and transistor resistances (*r*_*T*_) but are generally dimensionless apart from time variables (e.g., τ_*T*_,τ_*N*_). The advantage of these models is that they are computationally less expensive to use than full SPICE simulations while preserving the crucial device and system characteristics.

### 2.1. Autonomous Behavioral Model: Design 1

The autonomous circuit behavior of design 1 can be explained by slightly modifying the two equations (Equations 1a,b) stated in section 1. The fluctuating resistance of the low barrier nanomagnet-based MTJ is represented by a correlated random number *r*_*MTJ*_ with values between −1 and +1 and an average dwell time of the fluctuation denoted by τ_*N*_. The NMOS transistor tunable resistance is denoted by *r*_*T*_ and the inverter is represented by a *sgn* function. Thus, the normalized output *m*_*i*_ = *V*_*OUT, i*_/*V*_*DD*_ of the *i*_*th*_ p-bit can be expressed as:

(2) $$\begin{array}{r} m_{i}\left(t+\Delta t\right)=\text{sgn}\left(r_{T,i}\left(t+\Delta t\right)-r_{MTJ,i}\left(t+\Delta t\right)\right) \end{array}$$

where Δ*t* is the simulation time step, *r*_*T, i*_ represents the NMOS transistor resistance tunable by the normalized input *I*_*i*_ = *V*_*IN, i*_/*V*_0_ (compare Equation 1a) where *V*_0_ is a fitting parameter which is ≈50mV for the chosen parameters and transistor technology (compare Figure 2B) and *r*_*MTJ, i*_ is a correlated random number generator with an average retention time of τ_*N*_. For design 1, the transistor represents the tunable component that works in conjunction with the unbiased stochastic signal of the MTJ. *r*_*T, i*_ as a function of input *I*_*i*_ is approximated by a tanh function with a response time denoted by τ_*T*_ modeled by the following equations:

(3) $$\begin{array}{r} r_{T,i}\left(t+\Delta t\right)=r_{T,i}\left(t\right)\text{exp}\left(-\Delta t/\tau_{T}\right)\\ \;+\left(1-\text{exp}\left(-\Delta t/\tau_{T}\right)\right)\left(\text{tanh}\left(I_{i}\left(t+\Delta t\right)\right)\right) \end{array}$$

where it can be clearly seen that the dimensionless quantity *r*_*T, i*_ representing the transistor resistance is bounded by −1 ≤ *r*_*T,i*_ ≤ 1 for all synaptic inputs. Figure 2B shows by utilizing SPICE simulation how *I*_*i*_ influences the average output *m*_*i*_ and shows that the average response of the circuit is in good agreement with the tanh-function used in Equation (3).

The synapse delay τ_*S*_ in computing the input *I*_*i*_ can be modeled by:

(4) $$\begin{array}{r} I_{i}\left(t+\Delta t\right)=I_{i}\left(t\right)\text{exp}\left(-\Delta t/\tau_{S}\right)\\ \;+\left(1-\text{exp}\left(-\Delta t/\tau_{S}\right)\right)I_{0}(\underset{j}{\sum }J_{ij}m_{j}\left(t\right)+h_{j})) \end{array}$$

For calculating *r*_*MTJ, i*_, at time *t*+Δ*t* a new random number will be picked according to the following equations:

(5a) $$\begin{array}{r} r_{flip,i}\left(t+\Delta t\right)=\text{sgn}\left(\exp\left(-\frac{\Delta t}{\tau_{N}}\right)-\text{ran}{\text{d}}_{\left[0,1\right]}\right) \end{array}$$

where rand_[0, 1]_ is a uniformly distributed random number between 0 and 1 and τ_*N*_ represents the average retention time of the fluctuating MTJ resistance. If *r*_*flip*_ is -1, a new random *r*_*MTJ*_ will be chosen between −1 and +1. Otherwise, the previous *r*_*MTJ*_(*t*) will be kept in the next time step (*t*+Δ*t*), which can be expressed as

(5b) $$\begin{array}{r} r_{MTJ,i}\left(t+\Delta t\right)=\frac{r_{flip,i}\left(t+\Delta t\right)+1}{2}r_{MTJ,i}\left(t\right)\\ \;-\frac{r_{flip,i}\left(t+\Delta t\right)-1}{2}\text{ran}{\text{d}}_{\left[-1,1\right]} \end{array}$$

where −1 ≤ *r*_*MTJ,i*_(*t*) ≤ 1.

The charge current flowing through the MTJ branch of p-bit design 1 can get polarized by the fixed layer of the MTJ and generate a spin current *I*_*s*_ that can tune/pin the MTJ dynamics by modifying τ_*N*_. This effect is needed for tuning the output of design 2 but is not desired in design 1. However, the developed behavioral model can account for this pinning effect according to

(6) $$\begin{array}{r} \tau_{N}=\tau_{N}^{0}\text{exp}\left(r_{MTJ}I_{MTJ}\right), \end{array}$$

where $\tau_{N}^{0}$ is the retention time of *r*_*MTJ*_ when *I*_*MTJ*_ = 0. The dimensionless pinning current *I*_*MTJ*_ is defined as *I*_*MTJ*_ = *I*_*s*_/*I*_*s*, 0_ where *I*_*s*, 0_ can be extracted by following the procedure of Figure 2F. This pinning effect by *I*_*MTJ*_ is much smaller in in-plane magnets (IMA) than perpendicular magnets (PMA) (Hassan et al., 2019) and is ignored for design 1 throughout this paper.

Figures 2A–D shows the comparison of this behavioral model for p-bit design 1 with SPICE simulation of the actual hardware in terms of fluctuation dynamics, sigmoidal characteristic response, autocorrelation time (τ_*corr*_), and step response time (τ_*step*_) and in all cases the behavioral model closely matches SPICE simulations. The SPICE simulation involves experimentally benchmarked modules for different parts of the device. The SPICE model for the s-MTJ model solves the stochastic Landau–Lifshitz–Gilbert equation for the LBM physics. For the transistors, 14 nm Predictive Technology Model^1^ is used. As simulator *HSPICE* is utilized with the .trannoise function and a time step of 1 ps. The simulating framework was benchmarked experimentally and by using standard simulation tools in the field (Datta, 2012; Torunbalci et al., 2018). The autonomous behavioral model for design 1 is labeled as “PPSL: design 1.” The benchmarking is done for two different LBMs: (1) Faster fluctuating magnet 1 with saturation magnetization *M*_*s*_ = 1100 emu/cc, diameter *D* = 22 nm, thickness *th* = 2 nm, in-plane easy axis anisotropy *H*_*k*_ = 1 Oe, damping coefficient α = 0.01, demagnetization field *H*_*d*_ = 4π*M*_*s*_ and (2) slower fluctuating magnet 2 with the same parameters as in magnet 1 except *D* = 150 nm. The supply voltage was set to *V*_*DD*_ = −*V*_*SS*_ = 0.4 V. The fast and slow fluctuations of the normalized output *m*_*i*_ = *V*_*OUT, i*_/*V*_*DD*_ are captured by changing the τ_*N*_ parameter in the PPSL model. In the steady-state sigmoidal response, *V*_0_ is a tanh fitting parameter that defines the width of the sigmoid and lies within the range of 40–60 mV reasonably well depending on which part of the sigmoid needs to be better matched. In Figure 2B, *V*_0_ value of 50 mV is used to fit the sigmoid from SPICE simulation. The following parameters have been extracted from the calibration shown in Figure 2, where Δ*t* = 1 ps was used: τ_*N*_ = 150 ps (magnet 1), τ_*N*_ = 1.5 ns (magnet 2), τ_*T*_ = 3 ps, *I*_*s*, 0_ = 120 μA (magnet 1), *I*_*s*, 0_ = 1 mA (magnet 2).

There are two types of time responses: (1) Autocorrelation time under zero input condition labeled as τ_*corr*_ and (2) step response time τ_*step*_. The full width half maximum (FWHM) of the autocorrelation function of the fluctuating output under zero input is defined by τ_*corr*_, which is proportional to the retention time τ_*N*_ of the LBM. The step response time τ_*step*_ is obtained by taking an average of the p-bit output over many ensembles when the input *I*_*i*_ is stepped from a large negative value to zero at time *t* = 0 and measuring the time it takes for the ensemble averaged output to reach its statistically correct value consistent with the new input. τ_*step*_ defines how fast the first statistically correct sample can be obtained after the input is changed. For p-bit design 1, τ_*step*_ is independent of LBM retention time τ_*N*_ and is defined by the NMOS transistor response time τ_*T*_, which is much faster (few picoseconds) than LBM fluctuation time τ_*N*_. The effect of these two very different time scales in design 1 (τ_*step*_ ≪ τ_*corr*_) on an autonomous BN is described in section 3.

### 2.2. Autonomous Behavioral Model: Design 2

The autonomous behavioral model for design 2 is proposed in Sutton et al. (2020). In this article, we have benchmarked this model with the SPICE simulation of the single p-bit steady state and time responses shown in Figures 2E–H. According to this model, the normalized output *m*_*i*_ = *V*_*OUT, i*_/*V*_*DD*_ can be expressed as:

(7a) $$\begin{array}{r} m_{i}\left(t+\Delta t\right)=m_{i}\left(t\right)\text{sgn}(p_{NOTflip,i}\left(t+\Delta t\right)-\text{ran}{\text{d}}_{\left[0,1\right]}) \end{array}$$

(7b) $$\begin{array}{r} p_{NOTflip,i}\left(t+\Delta t\right)=\exp(-\frac{\Delta t}{\tau_{N}\exp\left(I_{i}m_{i}\left(t\right)\right)}) \end{array}$$

where *p*_*NOTflip, i*_(*t*+Δ*t*) is the probability of retention of the *i*^*th*^ p-bit (or “not flipping”) in the next time step that is a function of average neuron flip time τ_*N*_, input *I*_*i*_, and the current p-bit output *m*_*i*_(*t*). Figure 2 shows how this simple autonomous behavioral model for design 2 matches reasonably well with SPICE simulation of the device in terms of fluctuation dynamics (Figure 2E), sigmoidal characteristic response (Figure 2F), autocorrelation time (τ_*corr*_) (Figure 2G), and step response time (τ_*step*_) (Figure 2H). In design 2, τ_*step*_ and τ_*corr*_ are both proportional to LBM fluctuation time τ_*N*_ unlike design 1.

Different time scales in p-bit designs 1 and 2 are also reported in Hassan et al. (2019) in an energy-delay analysis context. In this article, we explain the effect of these time scales in designing an autonomous BN (section 3).

## 3. Difference Between Designs 1 and 2 in Implementing Bayesian Networks

**Figure 3:**
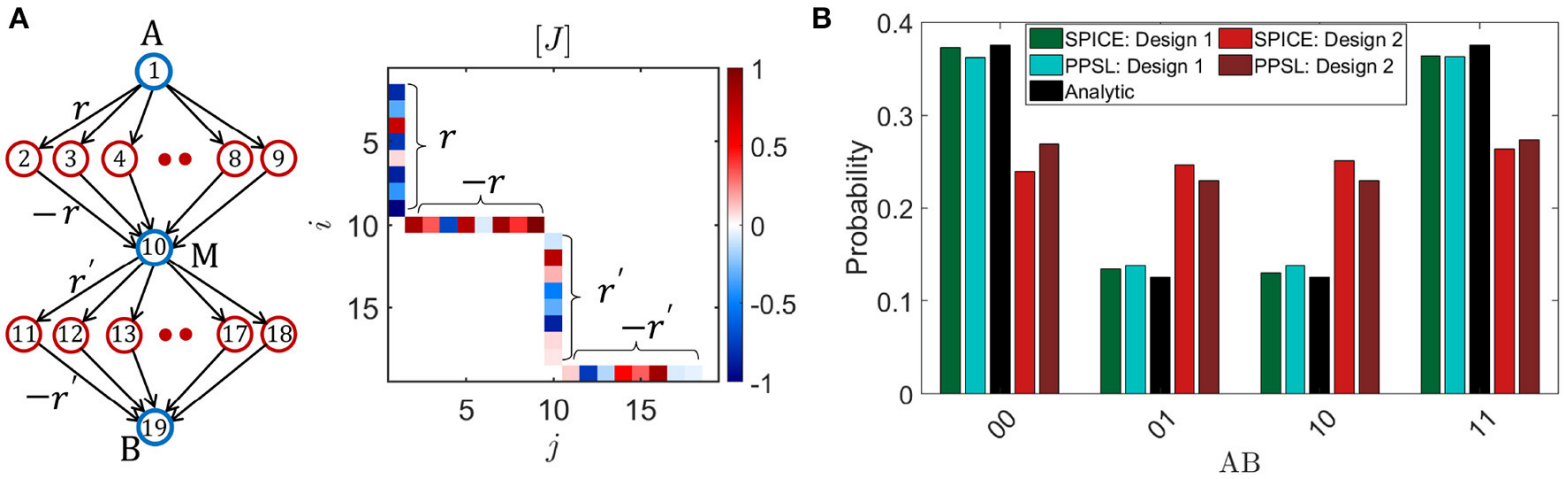
Difference between designs 1 and 2: **(A)** The behavioral models described in Figure 2 are applied to simulate a 19 p-bit BN with random *J*_*ij*_ between +1 and -1. The indices *i* and *j* of *J*_*i, j*_ correspond to the numbers inside each circle. The interconnections are designed in such a way so that pairs of intermediate nodes (*A, M*_1_) and (*M*_1_, *B*) get anti-correlated and (*A, B*) gets positively correlated. **(B)** The probability distribution of four configurations of *AB* are shown in a histogram from different approaches (SPICE, behavioral model and analytic). The behavioral models for two designs (labeled as PPSL) match reasonably well with the corresponding results from SPICE simulation of the actual hardware. Note that while design 1 matches with the standard analytical values quite well, design 2 does not works as an autonomous BN in general. For each histogram, 10^6^ samples have been collected.

The behavioral models introduced in section 2 are applied to implement a multi-layer belief/BN with 19 p-bits and random interconnection strengths between +1 and −1 (Figure 3A). For illustrative purposes, the interconnections are designed in such a way that although there are no meaningful correlations between the blue and red colored nodes with random couplings, pairs of intermediate nodes (*A, M*_1_) and (*M*_1_, *B*) get negatively correlated because of a net −*r*^2^ type coupling through each branch connecting the pairs. So it is expected that the start and end nodes (*A, B*) get positively correlated. Figure 3B shows histograms of four configurations (00, 01, 10, 11) of the pair of nodes *A* and *B* obtained from different approaches: Bayes rule (labeled as Analytic), SPICE simulation of design 1 (SPICE: Design 1) and design 2 (SPICE: Design 2), and autonomous behavioral model for design 1 (PPSL: Design 1) and design 2 (PPSL: design 2). It is shown that results from SPICE simulation and behavioral model for design 1 matches reasonably well with the standard analytical values showing 00 and 11 states with highest probability, whereas design 2 autonomous hardware does not work well in terms of matching with the analytical results and shows approximately all equal peaks. We have tested this basic conclusion for other networks as well with more complex topology as shown in Supplementary Figure 1. The analytical values are obtained from applying the standard joint probability rule for BNs (Pearl, 2014; Russell and Norvig, 2016), which is:

(8) $$\begin{array}{r} P\left(x_{1},x_{2},\ldots ,x_{N}\right)=\overset{N}{\underset{i=1}{\prod }}P(x_{i}|Parents\left(x_{i}\right)) \end{array}$$

Joint probability between two specific nodes *x*_*i*_ and *x*_*j*_ can be calculated from the above equation by summing over all configurations of the other nodes in the network, which becomes computationally expensive for larger networks. But one major advantage of our probabilistic hardware is that probabilities of specific nodes can be obtained by looking at the nodes of interest ignoring all other nodes in the system similar to what Feynman stated about a probabilistic computer imitating the probabilistic laws of nature (Feynman, 1982). Indeed, in the BN example in Figure 3, the probabilities of different configurations of nodes *A* and *B* were obtained by looking at the fluctuating outputs of the two nodes ignoring all other nodes. For the SPICE simulation of design 1 hardware, tanh fitting parameter *V*_0_ = 57 mV is used and the mapping principle from dimensionless coupling terms *J*_*ij*_ to the coupling resistances in the hardware is described in Faria et al. (2018). An example of this mapping is given in the Supplementary Material.

The reason why design 1 works for a BN and design 2 does not is because of the two very different time responses of the two designs shown in Figure 2 due to the fact that the tunable component is the transistor in design 1 (τ_*step*_∝τ_*T*_) and the MTJ in design 2 (τ_*step*_∝τ_*N*_). It is these two different time scales in design 1 (τ_*step*_ ≪ τ_*corr*_) that naturally ensures a parent to child informed update order in a BN. The reason is that when τ_*step*_ is small, each child node can immediately respond to any change of its parent nodes that happens due to a random event, which have a much larger time scale ∝τ_*corr*_. Thus, due to that fast step response, information about changing p-bits at the parent node can propagate quickly through the network and the output of the child nodes can be conditionally satisfied with the parent nodes very fast. Otherwise, if τ_*corr*_ gets comparable to τ_*step*_, the child nodes will not be able to keep up with the fast changing parent nodes since the information of the parent p-bit state has not been propagated through the network. As a result, the child nodes will produce a substantial number of statistically incorrect samples over the entire time range, thus deviating from the correct probability distribution. This effect is especially strong for networks where the coupling strength between p-bits is large.

**Figure 4:**
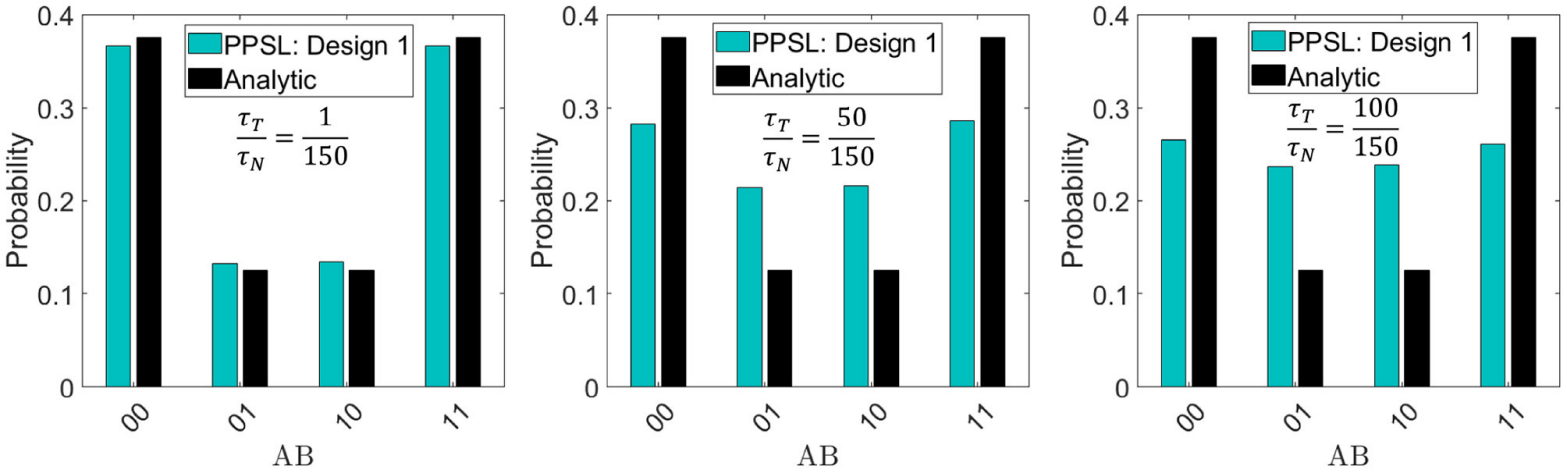
Effect of step response time in design 1: The reason for design 1 to work accurately as an autonomous Bayesian network as shown in Figure 3 is the two different time scales (τ_*T*_ and τ_*N*_) in this design with the condition that τ_*T*_ ≪ τ_*N*_. The same histogram shown in Figure 3 is plotted using the proposed behavioral model for different τ_*T*_/τ_*N*_ ratios and compared with the analytical values. It can be seen that as τ_*T*_ gets comparable to τ_*N*_, the probability distribution diverges from the standard statistical values. For each histogram 10^6^, samples have been collected.

To illustrate this point, the effect of τ_*step*_/τ_*corr*_ ratio is shown in Figure 4 for the same BN presented in Figure 3 by plotting the histogram of *AB* configurations for different τ_*T*_/τ_*N*_ ratios. It is shown that when τ_*T*_/τ_*N*_ ratio is small, the histogram converges to the correct distribution. As τ_*T*_ gets comparable to τ_*N*_, the histogram begins to diverge from the correct distribution. Thus, the very fast NMOS transistor response in design 1 makes it suitable for an autonomous BN hardware. One thing to note that under certain conditions, results from design 2 can also match the analytical results if spin current bias is large enough to drive down the fast step response time to ensure τ_*step*_ ≪ τ_*corr*_.

So apart from ensuring a fast synapse compared to neuron fluctuation time (τ_*S*_ ≪ τ_*N*_), which is the design rule for an autonomous probabilistic hardware, the autonomous BN demands an additional p-bit design rule that is a much faster step response time of the p-bit compared to its fluctuation time (τ_*step*_ ≪ τ_*N*_) as ensured in design 1. In all the simulations, the LBM was a circular in-plane magnet whose magnetization spans all values between +1 and −1 and negligible pinning effect. If the LBM is a PMA magnet with bipolar fluctuations having just two values +1 and −1, design 1 will not provide any sigmoidal response except with substantial pinning effect (Borders et al., 2019). Under this condition, τ_*step*_ of design 1 will be comparable to τ_*N*_ again and the system will not work as an autonomous BN in general. Therefore, LBM with continuous range fluctuation is expected for design 1 p-bit to work properly as a BN.

## 4. Discussion

In this article, we have elucidated the design criteria for an autonomous clockless hardware for BNs that requires a specific parent to child update order when implemented on a probabilistic circuit. By performing SPICE simulations of two autonomous probabilistic hardware designs built out of p-bits (designs 1 and 2 in Figure 1), we have shown that the autonomous hardware will naturally ensure a parent to child informed update order without any sequencers if the step response time (τ_*step*_) of the p-bit is much smaller than its autocorrelation time (τ_*corr*_). This criteria of having two different time scales is met in design 1 as τ_*step*_ comes from the NMOS transistor response time τ_*T*_ in this design, which is few picoseconds. We have also proposed an autonomous behavioral model for design 1 and benchmarked it against SPICE simulation of the actual hardware. All the simulations using behavioral model for design 1 are performed ignoring some non-ideal effects listed as follows:

- Pinning of the s-MTJ fluctuation due to STT effect is ignored by assuming *I*_*MTJ*_ = 0 in Equation (6). This is a reasonable assumption considering circular in-plane magnets that are very difficult to pin due to the large demagnetization field that is always present, irrespective of the energy barrier (Hassan et al., 2019). This effect is more prominent in perpendicular anisotropy magnets (PMA) magnets. It is important to include the pinning effect in p-bits with bipolar LBM fluctuations because in this case the p-bit does not provide a sigmoidal response without the pinning current. This effect is also experimentally observed in Borders et al. (2019) for PMA magnets. Such a p-bit design with bipolar PMA and STT pinning might not work for BNs in general, because in this case τ_*step*_ will be dependent on magnet fluctuation time τ_*N*_.
- In the proposed behavioral model, the step response time of the NMOS transistor τ_*T*_ in design 1 is assumed to be independent of the input *I*. But there is a functional dependence of τ_*T*_ on *I* in real hardware.
- The NMOS transistor resistance *r*_*T*_ is approximated as a tanh function for simplicity. In order to capture the hardware behavior in a better way, the tanh can be replaced by a more complicated function and the weight matrix [*J*] will have to be learnt around that function.

All the non-ideal effects listed above are supposed to have minimal effects on different probability distributions shown in this article. Real LBMs may suffer from common fabrication defects, resulting in variations in average magnet fluctuation time τ_*N*_ (Abeed and Bandyopadhyay, 2019). The autonomous BN is also quite tolerant to such variations in τ_*N*_ as long as τ_*T*_ ≪ *min*(τ_*N*_).

It is important to note that, for design 1 (Transistor-controlled) to function as a p-bit that has a step response time (τ_*step*_) much smaller than its average fluctuation time (τ_*N*_), the LBM fluctuation needs to be continuous and not bipolar. It is important to note that while most experimental implementations of low barrier magnetic tunnel junctions or spin-valves exhibit telegraphic (binary) fluctuations (Pufall et al., 2004; Locatelli et al., 2014; Parks et al., 2018; Debashis et al., 2020), theoretical results (Abeed and Bandyopadhyay, 2019; Hassan et al., 2019; Kaiser et al., 2019) indicate that it should be possible to design low barrier magnets with continuous fluctuations. Preliminary experimental results for such circular disk nanomagnets have been presented in Debashis et al. (2016). We believe that a lack of experimental literature on such magnets is partly due to the lack of interest of randomly fluctuating magnets that have long been discarded as impractical and irrelevant. The other experimentally demonstrated p-bits (Ostwal et al., 2018; Ostwal and Appenzeller, 2019; Debashis, 2020) fall under design 2 category with the LBM magnetization tuned by SOT effect and are not suitable for autonomous BN operation in general. It might also be possible to design p-bits using other phenomena such as voltage controlled magnetic anisotropy (Amiri and Wang, 2012), but this is beyond the scope of the present study. Here, we have specifically focused on two designs that can be implemented with existing MRAM technology based on STT and SOT.

## Data Availability Statement

The original contributions presented in the study are included in the article/Supplementary Material, further inquiries can be directed to the corresponding author/s.

## Author Contributions

RF and SD wrote the paper. RF performed the simulations. JK helped setting up the simulations. KC developed the simulation modules for the BSN in SPICE. All authors discussed the results and helped refine the manuscript.

## Conflict of Interest

The authors declare that the research was conducted in the absence of any commercial or financial relationships that could be construed as a potential conflict of interest.

## Footnotes

^1^http://ptm.asu.edu/
