# Supplementary material for: Hardware Design for Autonomous Bayesian Networks
Source: Front Comput Neurosci. 2021 Mar 8;15:584797. doi: 10.3389/fncom.2021.584797 (PMC7982658; doi:10.3389/fncom.2021.584797)
Supplement: Supplementary file 1 [file Data_Sheet_1.PDF]

Supplementary Material

1 MAPPING OF BAYESIAN NETWORKS TO HARDWARE P-CIRCUITS

To illustrate how Bayesian networks can be built with p-bits, in this section the mapping from a conditional probability table (CPT) to a hardware p-circuit is shown in a small example which was first proposed by Faria et al. (2018). As example, we use a 3 p-bit Bayesian network of Fig. 1 in the main manuscript which is also shown in fig. S1. Here, p-bit m_1 has no input which means that $P(m_1 = 1) = P(m_1 = -1) = p_1 = 0.5$. Because m_1 is parent of m_2, the state of m_2 will depend on the state of m_1. The dependence can be written in a CPT

	$m_1 = -1$	$m_1 = 1$
$m_2 = -1$	p_2	$1 - q_2$
$m_2 = 1$	$1 - p_2$	q_2

where for example p_2 is the probability that $m_2 = 1$ when $m_1 = 1$. The process can be performed analog for p-bits m_2 and m_3:

	$m_2 = -1$	$m_2 = 1$
$m_3 = -1$	p_3	$1 - q_3$
$m_3 = 1$	$1 - p_3$	q_3

The joint probability distribution can now be written as

$$P(m_1, m_2, m_3) = P(m_1)P(m_2|m_1)P(m_3|m_2) \quad \text{(S1)}$$

where for example $P(m_2|m_1) = p_2$ if $m_1 = -1$ and $P(m_2|m_1) = 1 - q_2$ if $m_1 = 1$.

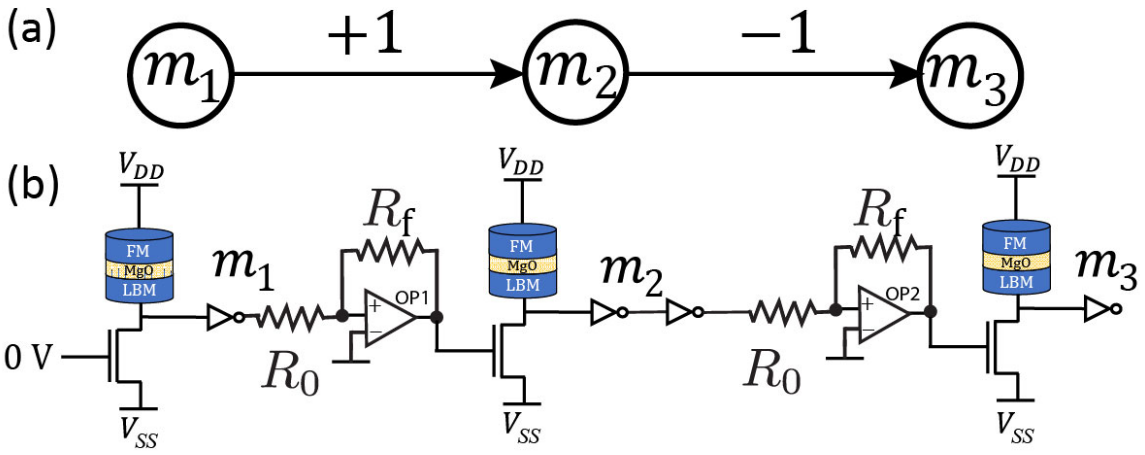

Figure S1. Example Bayesian network and hardware equivalent a) 3 node Bayesian network with. b) hardware mapping of (a).

As a next step p_2, q_2, $p3$ and q_3 have to be converted to the p-bit input-output characteristic which can be described by a tanh-function as shown in Fig. 2b of the main manuscript. This process is outlined in detail in Faria et al. (2018). For the connection between m_1 and m_2 which is defined by the matrix element $J_{2,1}$ we obtain (compare Eq. 1b of main manuscript)

$$J_{2,1} = \tanh^{-1}(2p_2 - 1). \tag{S2}$$

where we have made use of the fact that the bias $h_2 = 0$ which results in $p_2 = q_2$. Analog, for $J_{3,2}$ with $p_3 = q_3$ we get

$$J_{3,2} = \tanh^{-1}(2p_3 - 1). \tag{S3}$$

where we set $I_0 = 1$. To connect back to fig. S1 we want $J_{2,1} = 1$ and $J_{3,2} = -1$. This means in our example $p_2 = 1 - p3 = (\tanh(1) + 1)/2 \approx 0.88$.

To map the dimensionless system fig. S1a to the hardware circuit of fig. S1b, the connection matrix has to be mapped to resistances and the p-bit input and outputs to voltages (following Camsari et al. (2017)). Since the input of p-bit m_1 is 0 V, the output voltage $V_{OUT,1}$ will fluctuate between V_{DD} and $V_{SS} = -V_{DD}$ with a 50% probability of both states. For the input voltage of p-bit m_2 $V_{IN,2}$, we obtain the following expression using the current adder formula for an operational amplifier $V_{IN,2} = m_1 \frac{V_{DD}}{R_0} R_f$. The input of p-bit m_2 is scaled by V_0 (compare fig.2b of the main manuscript). To achieve a coupling of $J_{2,1}$, we want

$$J_{2,1} = \frac{V_{DD}}{R_0} \frac{R_f}{V_0} = 1 \tag{S4}$$

in our example. Since the voltages are fixed, the resistances have to be chosen to satisfy eq. S4. Depending on the sign of $J_{i,j}$, another inverter is needed as shown for p-bit m_2 since $J_{3,2} < 0$. Because $J_{2,1} = J_{3,2}$, the same resistance values can be used for both connections. In general, depending on the coupling matrix the resistances have to be chosen accordingly.

The p-bit histogram of network fig. S1a and b is shown in fig. 1d and e of the main manuscript, respectively.

2 ADDITIONAL BAYESIAN NETWORK EXAMPLE

The basic conclusion presented in fig. 3 of the main manuscript is tested for other networks as well with more complex topology. Figure S2 shows two more examples of Bayesian networks implemented on an autonomous hardware using two p-bit designs (design 1 and design 2) as shown in fig. 1 of the main manuscript. For both the examples, the probability distribution of four configurations of nodes A and B are shown in a histogram and compared with standard analytical results from applying probability chain rule. It is shown that results from design 1 autonomous hardware match well with the analytical results, but design 2 does not match. These two examples again varify the fact that design 1 autonomous hardware works for Bayesian networks in general, but design 2 does not.

REFERENCES

Camsari, K. Y., Salahuddin, S., and Datta, S. (2017). Implementing p-bits with embedded mtj. *IEEE Electron Device Letters* 38, 1767–1770

Faria, R., Camsari, K. Y., and Datta, S. (2018). Implementing bayesian networks with embedded stochastic mram. *AIP Advances* 8, 045101

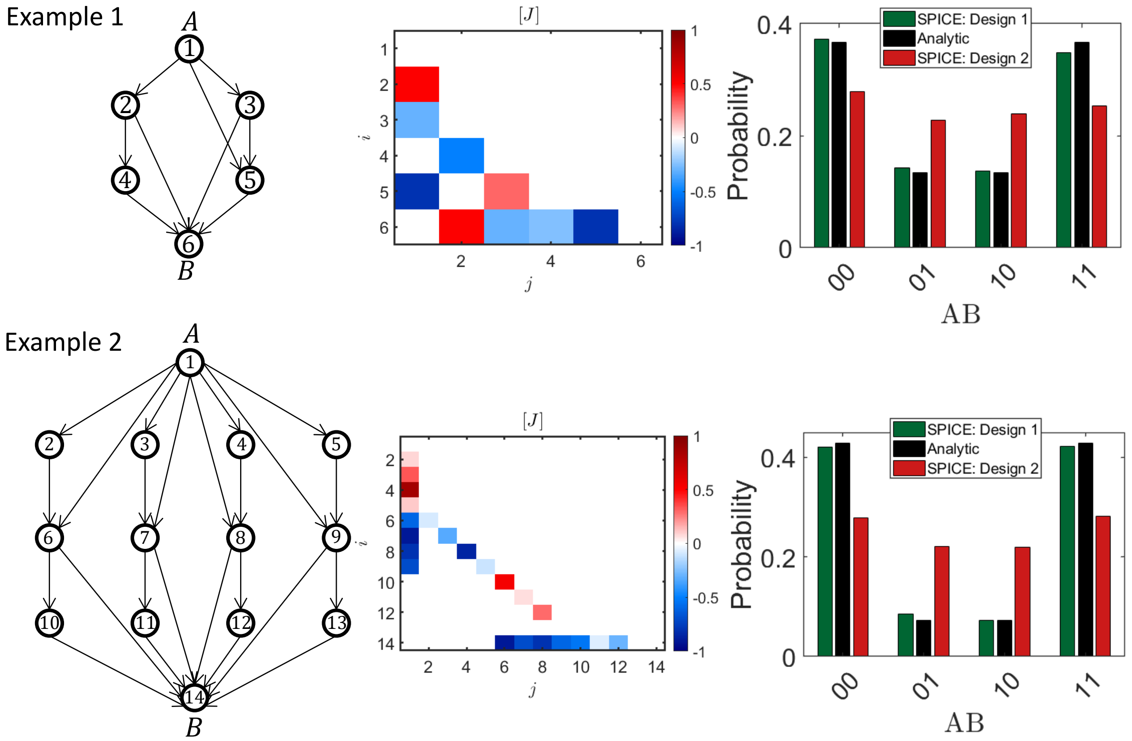

Figure S2. Difference between design 1 and design 2 for two different Bayesian network examples: Example 1 is a six node network with random interconnection strength between $+1$ and -1 as shown in the heatmap of the coupling matrix $[J]$. Example 2 is a larger network consisting of 14 nodes with random interconnection strength between $+1$ and -1. Both the networks have connections not only between two consecutive layers as in common multilayer perceptron type neural networks, but also connection from other layers. SPICE simulation of both the networks implemented on an autonomous hardware with design 1 and design 2 p-bits shows that probability distribution of four configurations of nodes (A, B) from design 1 matches the standard analytical results, but results from design 2 does not.
